# Supplementary material for: Identification of photoperiod-induced specific miRNAs in the adrenal glands of Sunite sheep (Ovis aries)
Source: Front Vet Sci. 2022 Jul 22;9:888207. doi: 10.3389/fvets.2022.888207 (PMC9354845; doi:10.3389/fvets.2022.888207)
Supplement: Supplementary file 1 [file Data_Sheet_1.ZIP › Supplementary file/miRDeep2 file.zip!/miRDeep2 file/Novel_Structure/SPLP42A3/Novel_232.pdf]

Provisional ID	:	NC_019458.2_3318
Score total	:	200.5
Score for star read(s)	:	3.9
Score for read counts	:	193.4
Score for mfe	:	1.7
Score for randfold	:	1.6
Score for cons. seed	:	
Total read count	:	391
Mature read count	:	389
Loop read count	:	0
Star read count	:	2

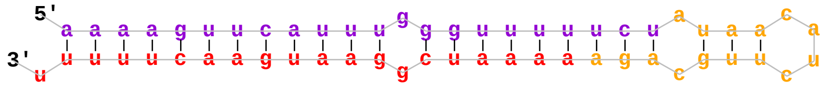

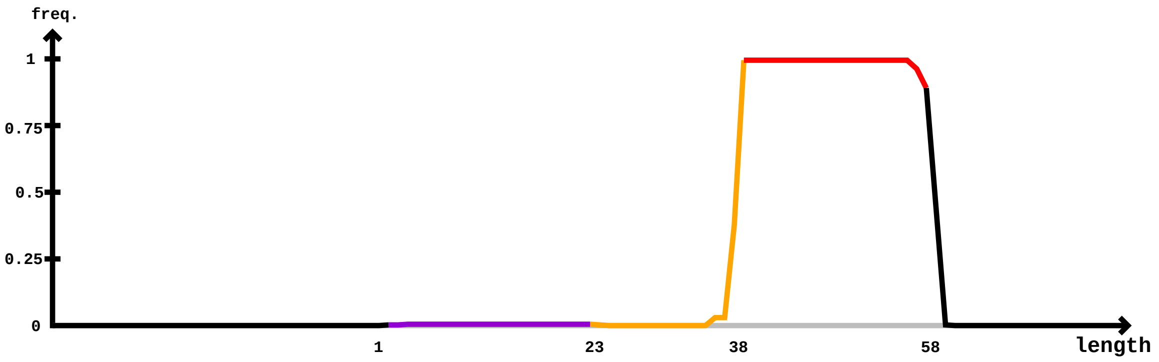

Star Mature

```
5'- ggaggagaugaguccagcguuuuggguugaccuaaaaguucauuuggguuuuucuauaacaucuugcagaaaaaucggaaugaacuuuuuggccuacccaauaaauaaaa   -3'    obs
    ggaggagaugaguccagcguuuuggguugaccuaaaaguucauuuggguuuuucuauaacaucuugcagaaaaaucggaaugaacuuuuuggccuacccaauaaauaaaa          exp
    (..(((......)))..)...((((((.(.((.((((((((((((.(((((((((.(((....))).))))))))).)))))))))))).)).).)))))).........   reads      mm         sample
    .................................aaaaguucauuAggguuuuucu.......................................................   1          1          seq
    ...................................aaguucauuuggguuuuucC.......................................................   1          1          seq
    ...................................................................agaaaaaucUgaaugaacuuu......................   1          1          seq
    ...................................................................agaaaaaucUgaaugaacuuuu.....................   3          1          seq
    ...................................................................agaaaaaucUgaaugaacuuuuu....................   8          1          seq
    .....................................................................aaaaaucUgaaugaacuuu......................   12         1          seq
    .....................................................................aaaaaucUgaaugaacuuuu.....................   26         1          seq
    .....................................................................aaaaaucUgaaugaacuuuuu....................   78         1          seq
    .....................................................................aaaaaucUgaaugaacuuuuug...................   24         1          seq
    ......................................................................aaaaucggaaugaacuuuuu....................   1          0          seq
    ......................................................................aaaaucUgaaugaacuuuuu....................   247        1          seq
    ......................................................................aaaaCcggaaugaacuuuuugg..................   1          1          seq
```
